# Identification of associated risk factors for serological distribution of hepatitis B virus via machine learning models

**DOI:** 10.1186/s12879-023-08911-8

**Published:** 2024-01-09

**Authors:** Ning Yao, Yang Liu, Jiawei Xu, Qing Wang, Quanhua Zhou, Yue Wang, Dong Yi, Yazhou Wu

**Affiliations:** 1 Department of Health Statistics, College of Preventive Medicine, Army Medical University, NO.30 Gaotanyan Street, Shapingba District, Chongqing, 400038 China; 2 Chongqing Center for Disease Control and Prevention, NO.8 Changjiang 2nd Street, Yuzhong District, Chongqing, 400042 China

**Keywords:** Hepatitis B, Machine learning, Sero-epidemiology, Vaccine

## Abstract

**Background:**

The provincial-level sero-survey was launched to learn the updated seroprevalence of hepatitis B virus (HBV) infection in the general population aged 1–69 years in Chongqing and to assess the risk factors for HBV infection to effectively screen persons with chronic hepatitis B (CHB).

**Methods:**

A total of 1828 individuals aged 1–69 years were investigated, and hepatitis B surface antigen (HBsAg), antibody to HBsAg (HBsAb), and antibody to B core antigen (HBcAb) were detected. Logistic regression and three machine learning (ML) algorithms, including random forest (RF), support vector machine (SVM), and stochastic gradient boosting (SGB), were developed for analysis.

**Results:**

The HBsAg prevalence of the total population was 3.83%, and among persons aged 1–14 years and 15–69 years, it was 0.24% and 4.89%, respectively. A large figure of 95.18% (770/809) of adults was unaware of their occult HBV infection. Age, region, and immunization history were found to be statistically associated with HBcAb prevalence with a logistic regression model. The prediction accuracies were 0.717, 0.727, and 0.725 for the proposed RF, SVM, and SGB models, respectively.

**Conclusions:**

The logistic regression integrated with ML models could helpfully screen the risk factors for HBV infection and identify high-risk populations with CHB.

## Background

The viral hepatitis pandemic has become a heavy toll on lives and a great burden on communities and health systems worldwide. It accounts for over 1.4 million deaths annually due to acute infection and related hepatic carcinoma and cirrhosis [1]. Hepatitis B virus (HBV) is the most frequent cause of acute hepatitis and chronic liver illness [2], with 1.5 million newly infected with chronic infection and 0.82 million dying from HBV-related diseases globally in 2019 [3]. Most people are unaware of their HBV infection because of its asymptomatic characteristics [4, 5]. Hepatitis B surface antigen (HBsAg) lasting for six months or longer is defined as chronic hepatitis (CHB), which mostly results in complications of cirrhosis and hepatocellular carcinoma (HCC) that are a major threat to human health [4].

China has long been a high prevalence area for HBV infection, with 9.8% of the population seropositive for HBsAg and approximately 60% having a history of HBV infection in 1992. HBV is a blood-borne infection, and comprehensive approaches are promoted to end hepatitis B epidemics, including vaccines, prevention of mother-to-child transmission (MTCT), promotion of safe injection and surgical practice, and securing the safety of blood products [4]. Currently, there are effective vaccines for preventing HBV with an immunization schedule of three doses administered within 24 h of birth and at one and six months of age. In China, the hepatitis B vaccine (HepB) was first recommended for infants to be routinely vaccinated in 1992, but parents should pay for both the vaccine and service [6]. Since 2002, HepB vaccination has been integrated into China’s national expanded program on immunization (EPI), and the vaccine was free for children to be routinely immunized [7]. The initial dose provided was 5 µg/0.5 mL, which was proven to be effective for the prevention of MTCT in 85–90% of children [8]. The HepB dose was further increased to 10 µg/0.5 mL in 2011 to improve the effectiveness of the program.

China conducted three nationwide serosurveys of HBV prevalence in 1992, 2006 and 2014. HBsAg positivity was 9.8% and 7.2% in the general population aged 1–59 years in 1992 and 2006 [6, 9], respectively, and HBsAg prevalence fell to 2.64% among the population aged 1–29 years in 2014 [10]. The results showed that the prevalence of HBsAg has decreased significantly in the population, including children under 5 years, decreasing from 9.67% in 1992 to 0.32% in 2014, which is already ahead of the 1% control target set by the Western Pacific Region of the World Health Organization (WHO) [1]. Chongqing is a municipality under the direct control of the central government located in southwest China. The serosurvey of 2006 and 2014 reported that 10.72% and 3.89% of the general population aged 1–59 years and 1–29 years, respectively, were positive for HBsAg, which was higher than the national level. The latest provincial-level serosurvey of the population aged 1–69 years was conducted in 2020 in two districts of Chongqing to explore the updated seroprevalence of HBV.

The susceptibility and immunity across various age groups for HBV infection have been greatly reshaped according to the national immunization program and other public health interventions [11]. Three main HBV serum markers, HBsAg, antibody to HBsAg (HBsAb), and antibody to B core antigen (HBcAb), are usually used to evaluate the effect of vaccination on preventing HBV infection in the general population [10]. The presence of HBsAb often denotes the existence of immunity toward HBV infection, obtained either by vaccination or resolved infection, while HBcAb is an indicator of natural HBV infection that can be detected in nearly all patients exposed to HBV [12]. An examination of the seroprevalence of the above three markers, together with related epidemiological factors in the general population, would offer up-to-date evidence for determining the potential risk of HBV transmission and assisting future vaccination designs. The objectives of the study are to learn the seroprevalence of HBV in the general population in Chongqing and to assess the risk factors for HBV transmission along with the influences of vaccination history. The expected evidence would assist in forming future public health interventions for HBV prevention and control.

## Methods

### Study population

The sample size was calculated based on the equation below, where *p* is the prevalence of HBsAg in those aged 1–29 years in Chongqing in the 2014 serosurvey (3.89%). $z_{\alpha/2}$ = 1.96, $\delta$ = 0.02 was the maximum allowed error, and *deff* was the design effect (2.25).

$$n = \left(\frac{z_{\alpha/2}^{2} \times p \times (1-p)}{\delta^2}\right)\times deff$$

Based on the equation, the primary sample size calculated was 808. Considering that the investigated population should be extended to 1–69 years this time, the sample size was set to 2.25-fold of 808, which was 1818. Two districts (Dazu and Wanzhou) located in eastern and northwestern Chongqing were selected according to the multistage sampling method of the national guidelines. Two towns or villages were randomly picked in each district representing two major regional groups: urban and rural areas. Residents aged 1–69 years who lived for more than 6 months were recruited using probability proportionate to size sampling (PPS) method according to age groups through the health clinics at the survey site. The health clinic in each village has a list of residents across different age groups, subjects aged 1–4, 5–14, 15–29, and 30–69 were enumerated and selected based on the systematic interval from the list.

### Investigation

After informed consent was acquired from subjects or legal guardians of children aged 1–14 years, face-to-face interviews were completed by trained staff based on a standard questionnaire collecting information such as demographic characteristics, immunization status, history of hepatitis, and knowledge of hepatic disease (for adults aged 15–69 years only). Children’s immunization information was documented from the Chongqing Immunization Planning Information Management System or immunization records kept by the parents. HepB immunization of subjects older than 15 years was recorded based on memory (vaccinated, unvaccinated, unknown). Three milliliter and 5 ml venous blood samples were obtained from children aged 1–14 years and adults aged above 15 years, respectively. Serum was separated in laboratories of the local Centers for Disease Control and Prevention (CDC) and then stored at -20 °C until transported to the laboratory of Chongqing CDC.

### Laboratory testing and statistical analysis

HBsAg, HBsAb, and HBcAb were detected using enzyme-linked immunosorbent assay (ELISA) reagents (Beijing WANTAI Biological Pharmacy Enterprise Co. Ltd.). Subjects aged 15–69 years with HBsAg-positive results were engaged in the further conduction of clinical examinations and liver biochemistry test. Data were double entered into EpiData version 3.1 (The EpiData Association, Odense, Denmark). Data analysis was conducted using R, version 4.0.1 (R Foundation for Statistical Computing, Vienna, Austria). Frequency/proportions were used to describe the distribution of HBV serological markers. The Chi-Square test was used for the comparison of HBV serological markers between different groups. A two-tailed *P* value < 0.05 was considered to be significant. Logistic regression and machine learning (ML) models (random forest/RF, support vector machine/SVM, stochastic gradient boosting/SGB) were used for high-risk factor selection and the identification of target populations who were at high risk of HBV infection.

## Results

A total of 1828 subjects participated in the research with consenting and sufficient serum samples for lab analysis. More female subjects (61.05%, 1116/1828) were involved than male subjects (38.95%, 712/1828). The HBsAg prevalence of the total population was 3.83% (70/1828), and among persons aged 1–14 years and 15–69 years, it was 0.24% (1/416) and 4.89% (69/1412), respectively. The prevalence of HBsAb was negatively associated with age group, and the highest prevalence was observed in the 1–4 years old group (77.48%). The positivity of all three HBV serological markers was significantly different across age groups (*P* < 0.01). The seropositive rate of HBsAg was significantly lower in subjects with a confirmed HepB vaccination history than in those with an unknown or no vaccination history (1.68%, 5.78%, and 5.56%, respectively, *P* < 0.01) (Table 1).

**Table 1:** HBV serological markers by region, gender, immunization history, and different age groups (n/%)

Category	HBsAg^a^		HBsAb^b^		HBcAb^c^		Total
	Positive	Negative	Positive	Negative	Positive	Negative	
Age groups							
1–4 years	1(0.66)	150(99.34)	117(77.48)	34(22.52)	43(28.48)	108(71.52)	151(8.26)
5–14 years	0(-)	265(100.00)	127(47.92)	138(52.08)	55(20.75)	210(79.25)	265(14.50)
15–69 years	69(4.89)	1343(95.11)	757(53.61)	655(46.39)	809(57.29)	603(42.71)	1412(77.24)
*x*^2^ (*P*)	18.95(< 0.01)		37.22(< 0.01)		148.60(< 0.01)		
Sex							
Male	29(4.07)	683(95.93)	374(52.53)	338(47.47)	323(45.37)	389(54.63)	712(38.95)
Female	41(3.67)	1075(96.33)	627(56.18)	489(43.82)	584(52.33)	532(47.67)	1116(61.05)
*x*^2^ (*P*)	0.188(0.665)		2.344(0.126)		8.434(< 0.01)		
Region							
Urban	29(3.11)	902(96.89)	552(59.29)	379(40.71)	504(54.14)	427(45.86)	931(50.93)
Rural	41(4.57)	856(95.43)	449(50.06)	448(49.94)	403(44.93)	494(55.07)	897(49.07)
*x*^2^ (*P*)	2.63(0.105)		15.73(< 0.01)		15.49(< 0.01)		
Immunization history							
Yes	14(1.68)	818(98.32)	462(55.53)	370(44.47)	267(32.09)	565(67.91)	832(45.51)
No	40(5.56)	679(94.44)	399(55.49)	320(44.51)	477(66.34)	242(33.66)	719(39.33)
Unknown	16(5.78)	261(94.22)	140(50.54)	137(49.46)	163(58.84)	114(41.16)	277(15.15)
*x*^2^ (*P*)	19.132(< 0.01)		2.34(0.310)		192.12(< 0.01)		
Total	70(3.83)	1758(96.17)	1001(54.76)	827(45.24)	907(49.62)	921(50.38)	

Note: ^a^ HBsAg, hepatitis B surface antigen; ^b^ HBsAb, antibody to hepatitis B surface antigen; ^c^ HBcAb, antibody to hepatitis B core antigen

**Fig. 1:**
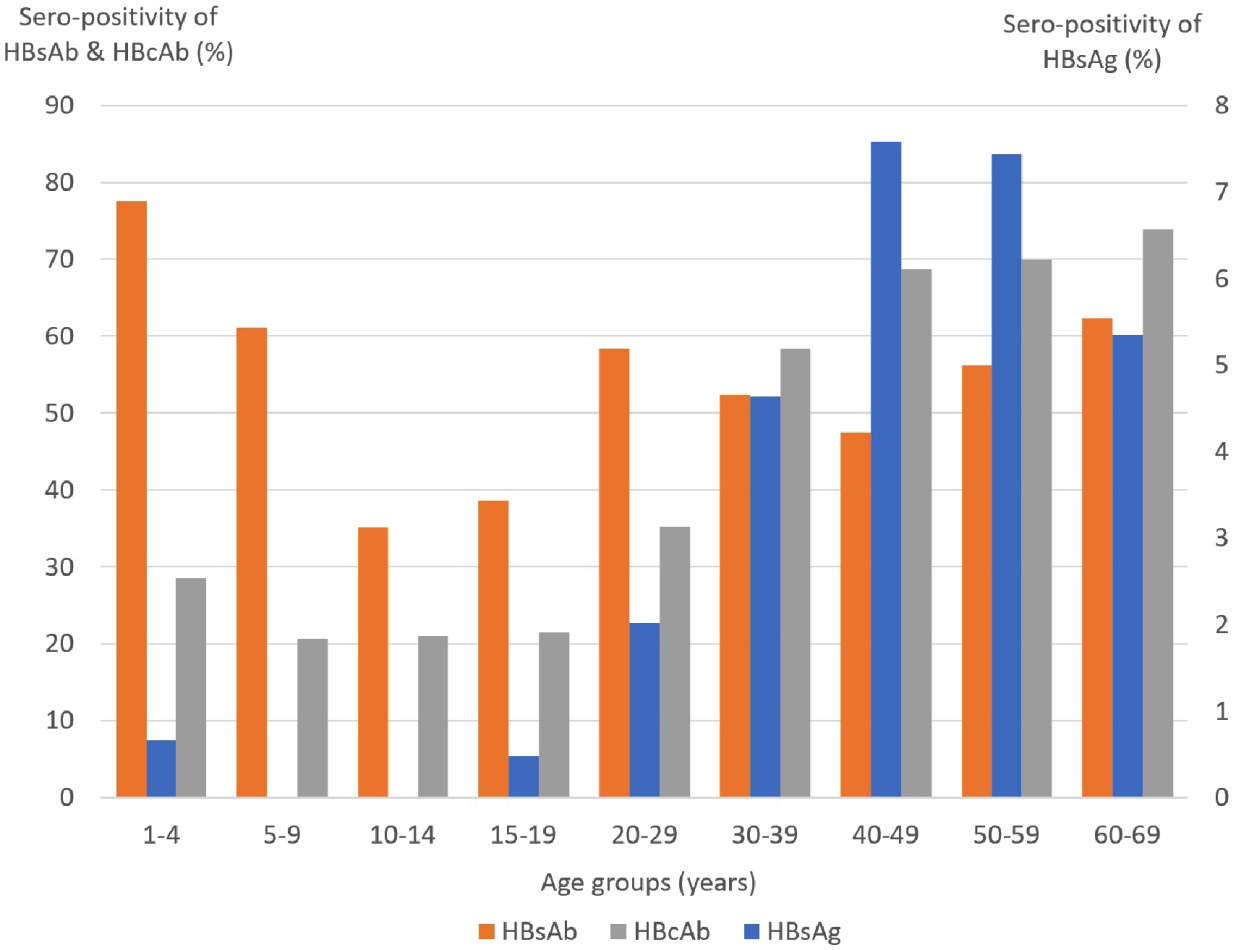
Sero-positivity of three HBV serological markers across different age groups. The prevailing trends of HBsAg and HBcAb were parallel across age groups, with lower percentages in younger children and higher percentages in elderly individuals, while the prevalence of HBsAb was lowest in the adolescent group. HBsAg, hepatitis B surface antigen; HBsAb, antibody to hepatitis B surface antigen; HBcAb, antibody to hepatitis B core antigen

The prevailing trend of HBsAg was lower among children aged 1–14 years and increased steadily from 0.48% of adolescents aged 15–19 years to 5.35% of elders aged 60–69 years with a peak of 7.58% of persons aged 40–49 years. The HBcAb seropositivity trend was similar to that of HBsAg and was highest among elderly individuals aged 60–69 years. The prevalence of HBsAb decreased with age from 77.48% of children aged 1–4 years to 35.07% of children aged 10–14 years and then increased with age to 62.26% of persons aged 60–69 years (Fig. 1).

### Immunization status and serological results of children under 15 years of age

According to the immunization documents of children under 15 years, 98.32% (409/416) were fully vaccinated with HepB, and 62.35% (255/412) of them were using 10 µg of HepB. All children aged 1–4 years (151) were fully vaccinated with HepB according to the immunization schedule. Five (5) mothers investigated were self-reported to be HBsAg-positive before delivery, and 4 of their children were vaccinated in a timely manner with one dose of 100 IU hepatitis B immune globulin (HBIG) and HepB (10 µg) at birth. The other subject was recorded to be unknown of the mother-to-child interruption performed at the delivery time. The only child tested to be HBsAg-positive was a girl aged 1 year and 8 months vaccinated with 3 doses of HepB, and her mother was recorded to be HBsAg-negative before delivery.

### History of hepatic disease and vaccination of adults aged 15–69 years

A total of 69 adults aged 15–69 years were HBsAg positive and 18 (26.09%) were women aged 15–49 years who were of childbearing age. The calculated prevalence of HBsAg in childbearing women was 3.70% (18/486). The educational level for subjects aged over 15 years was 13.74% (194/1412) for no formal education, 24.50% (346/1412) for primary school, 30.17% (426/1412) for junior high school, 15.72% (222/1412) for senior high school and 15.86% (224/1412) for college or higher. More than 3 quarters (79.71%, 55/69) of the subjects tested to be HBsAg-positive were previously HBsAg-negative or unknown. In addition, 57.29% (809/1412) of subjects were formerly infected with HBV, but 95.18% (770/809) of them were unaware of it. Self-recognition of previous HBV infection was not in accordance with the distribution of HBV serological markers (*P* < 0.01) (see Table 2).

**Table 2:** Relationships between serological markers and history of hepatic disease in adults aged 15–69 years (n/%)

Individual information	HBsAg^a^			HBcAb^b^		
	Positive	Negative	*x*^2^ (*P*)	Positive	Negative	*x*^2^ (*P*)
Education						
Illiteracy	12(6.19)	182(93.81)	6.52(0.16)	125(64.43)	69(35.57)	46.58(< 0.01)
Primary school	21(6.07)	325(93.93)		240(69.36)	106(30.64)	
Junior high school	22(5.16)	404(94.84)		235(55.16)	191(44.84)	
Senior high school	10(4.5)	212(95.50)		110(49.55)	112(50.45)	
College or higher	4(1.79)	220(98.21)		99(44.20)	125(55.80)	
Previously HBsAg-positive?						
Yes	14(20.29)	35(2.61)	62.65(< 0.01)	39(4.82)	10(1.66)	15.50(< 0.01)
No	44(63.77)	1127(83.92)		676(83.56)	495(82.09)	
Unknown	11(15.94)	181(13.48)		94(11.62)	98(16.25)	
Previously been diagnosed with fatty liver disease?						
Yes	6(8.70)	49(3.65)	3.22(0.07)^*^	38(4.70)	17(2.82)	3.25(0.07)
No	63(91.30)	1294(96.35)		771(95.30)	586(97.18)	
Previously been diagnosed with alcoholic hepatitis?						
Yes	0(-)	5((0.37)	0(1.00)^*^	4(0.49)	1(0.17)	0.33(0.565)^*^
No	69(100.00)	1338(99.63)		805(99.51)	602(99.83)	
Vaccinated with HepB						
Yes	13(18.84)	410(30.53)	4.28(0.1174)	169(20.89)	254(42.12)	79.64(< 0.01)
No	40(57.97)	672(50.04)		477(58.96)	235(38.97)	
Unknown	16(23.19)	261(19.43)		163(20.15)	114(18.91)	
Total	69(4.89)	1343(95.11)		809(57.29)	603(42.71)	

Note: ^a^ HBsAg, hepatitis B surface antigen; ^b^ HBsAb, antibody to hepatitis B surface antigen; *Continuity adjusted Chi-Square

### Clinical examination and liver biochemical tests of HBsAg-positive subjects

A total of 69 subjects aged 15–69 years were tested to be HBsAg positive, but 14 were refused to participate in the second-stage investigation or lost contact in the following months. Among the 55 follow-up subjects, ultrasound examination of the liver showed hepatomegaly in 3 cases, and echogenicity was uneven in one case and coarse in 8 cases. Thirteen (13) cases showed occupying liver lesions, 11 of which were cysts and 2 were hemangiomas.

Liver biochemical tests included albumin (reference range:40–55 g/L), globulin (ref:20–40 g/L), total bilirubin (ref: 5.1–28 µmol/L), alanine aminotransferase (ALT, ref: 7–40 U/L), and aspartate aminotransferase (AST, ref: 13–35 U/L). Only one subject had albumin < 40 g/L, two subjects had abnormal ALT (> 40 U/L), and a total of 6 subjects had AST > 35 U/L.

### Risk factors associated with HBcAb prevalence using machine learning models

Logistic regression was used to discover the influencing factors of HBcAb prevalence. The dependent variable was HBcAb seropositivity, and the independent variables were sex, region, immunization history, and age. The results showed that older people living in urban areas with an unvaccinated or unknown vaccination status were at higher risk of HBV infection than younger persons living in rural areas with a HepB vaccination history (Table 3).

**Table 3:** Multivariate logistic regression of HBcAb prevalence

Variable	Category	Parameter	Wald	aOR^a^ (95%CI)	*P*
Gender	Female	0.120	1.339	1.128(0.92–1.383)	0.247
Immunization history	Unvaccinated	1.150	80.642	3.157(2.46–4.604)	< 0.01
	Unknown	0.772	23.851	2.164(1.59–2.955)	< 0.01
Region	Urban	0.466	20.891	1.594(1.306–1.949)	< 0.01
Age	5–14 years	-0.423	3.185	0.655(0.412–1.045	0.074
	15–69 years	0.489	5.565	1.63(1.092–2.464)	0.018

^a^ aOR: adjusted odds ratios;

Three ML algorithms were applied to further inspect the relationship between demographic characteristics and seroprevalence of HBcAb. Although gender is not statistically associated with HBcAb prevalence in the logistic regression model, it is used for ML modeling referencing other similar literature [9]. Four demographic characteristics, including sex, region, age, and immunization history, were utilized as predictive features. The samples were divided into a training set (1280 subjects with 635 positives) and a testing set (548 subjects with 272 positives) based on a 7:3 ratio using the random sampling methodology. Three repeats of the 10-fold cross-validation were applied to the training set to build the ensemble classification models using the RF, SVM, and SGB methods. The SGB method has the highest values of area under the curve (AUC) and specificity over the other methods in the training set. Similar accuracy and high sensitivity were achieved across all three models in the validation process, with SGB on average performing better than the others (Table 4). DeLong AUC comparison methodology [13] was used to evaluate and compare the performance of 3 ML models in testing dataset. It compared the areas under two or more receiver operating characteristic curves (ROC) using nonparametric approach. The final test statistic estimate which follows a chi-square distribution was 5.22, P value was 0.07, indicated that the 3 considered ROC curves are equal, there was no significant difference between performances of the 3 ML models.

**Table 4:** High-risk population identification compared using three machine learning algorithms

Algorithm	Training dataset				Testing dataset				
	AUC	Sensitivity	Specificity		Sensitivity	Specificity	Accuracy	Kappa	F-measure
RF^a^	0.730	0.633	0.765		0.761	0.674	0.717	0.435	0.728
SVM^b^	0.741	0.686	0.718		0.728	0.725	0.727	0.453	0.725
SGB^c^	0.746	0.637	0.783		0.776	0.674	0.725	0.450	0.736

Note: ^a^ RF, random forest; ^b^ SVM, support vector machine; ^c^ SGB, stochastic gradient boosting;

**Fig. 2:**
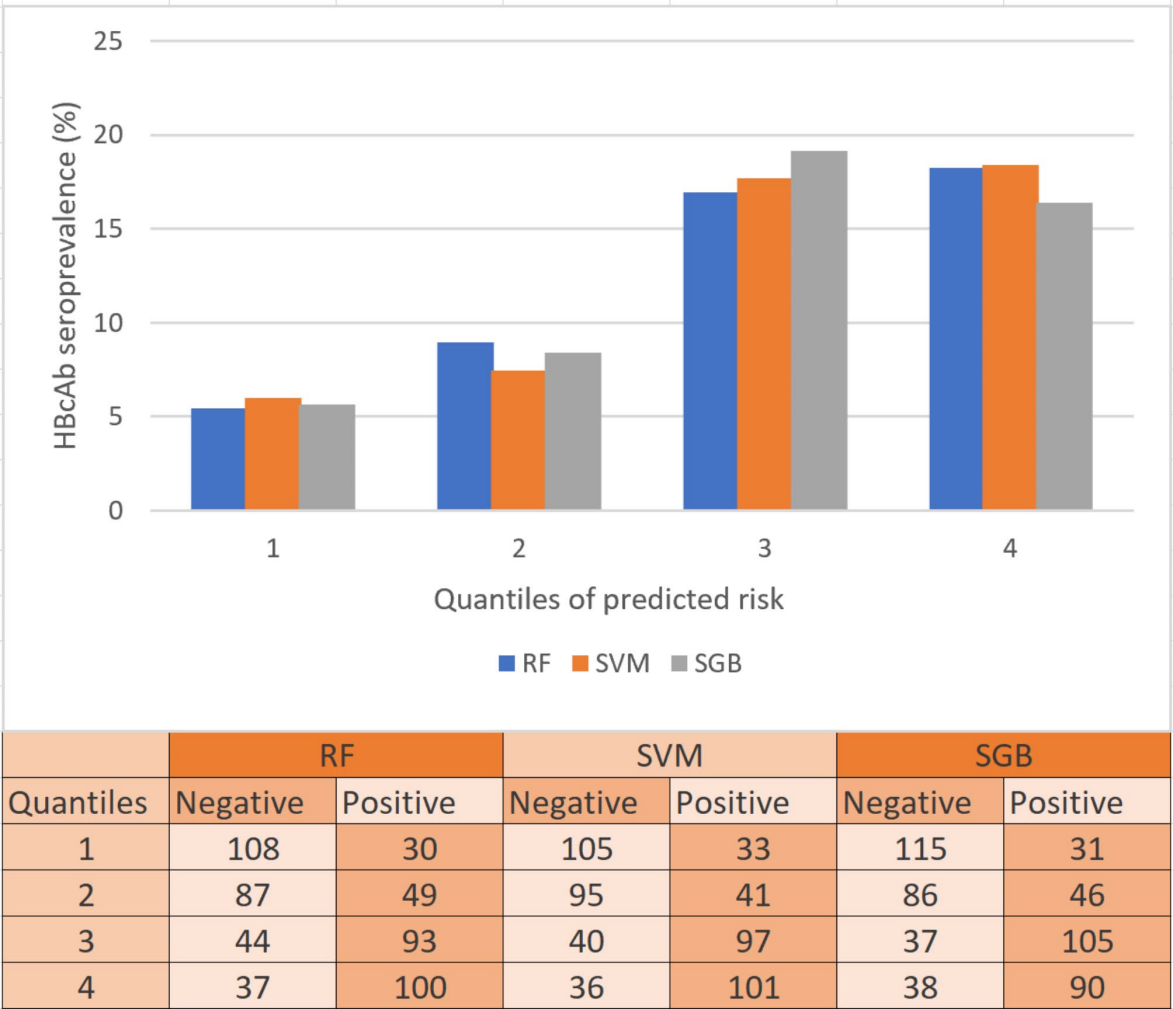
Percentage of subjects with HBV infection within each predicted risk quantile for the RF, SVM, and SGB models in the validation set. The percentage of subjects positive for HBcAb was higher in the upper quantiles than in the lower quantiles, and a comparison was conducted using the Chi-Square test. HBcAb, antibody to hepatitis B core antigen; RF, random forest; SVM, support vector machine; SGB, stochastic gradient boosting

The efficacy of the ML models was further assessed for differentiating subjects with a high likelihood of HBV infection from those with a low likelihood. Subjects were divided into quantile groups according to their probability of HBV infection predicted by three models [14]. The prevalence of HBcAb was higher in the 3^rd^ and 4^th^ quantiles of risk than in the lower two quantiles, and the percentage of subjects positive for HBcAb within each quantile was significantly different in all three models (Fig. 2).

## Discussions

Since the HepB was fully integrated into the EPI with emphasis on providing a timely birth dose for infants born within 24 h in 2002, a dramatic decrease in HBsAg prevalence among children aged 1–19 years has been observed along with a higher prevalence of HBsAb in this cross-sectional survey. Children in this age cohort have low infection risk because the HepB vaccination coverage and protection rate are estimated to be 95% and 98–100%, respectively [15]. The prevalence of HBsAb was slightly increased in the 20–29 year age group compared with the younger age group and then remained above 45% for subsequent ages, which could contribute to the booster dose of HepB administered to college freshmen with negative or weakly positive HBsAb [16, 17]. Persons aged 20–29 years also have a lower prevalence of HBsAg and HBcAb than those aged > 30 years because HepB was recommended for infant routine immunization in 1992, not for free [9], and the coverage of HepB was disequilibrium in different areas. Higher prevalences of both HBsAb and HBcAb were observed in subjects living in urban areas than in those living in rural areas, partly due to the imbalance in HepB coverage before the EPI program in 2002, and high-risk behaviors such as unsafe sexual behavior and blood transfusion or intravenous drug use were more common in urban areas [15]. Persons with a HepB vaccination history have a lower incidence of HBsAg positivity and natural infection across all age groups, indicating the proven efficiency of HepB in preventing HBV infection [18].

China has made every effort to curb the “vertical transmission” of HBV from mother to child at delivery time. It has been recommended to prevent MTCT of HBV infection by vaccination since plasma-derived HepB was available in China in 1986. Infants born to HBsAg-positive mothers received the first dose of HepB within 24 h after birth with HBIG, and completing the following 2 doses in subsequent months could reduce neonatal HBV infection by 85–95% [8]. Since 2012, China has launched a national program providing HBsAg, HIV, and syphilis screening for pregnant women and HBIG for infants born to HBsAg-positive mothers for free [3, 19]. With the efforts of timely birth dose vaccines of neonates and high 3-dose series coverage of HepB, both the prevalence of HBsAg in children under 5 years (0.66%) and childbearing women (3.70%) has greatly declined compared with the national level in 2006 (0.96% & 6.6%) [8].

The current situation of the high prevalence of active HBV infection among older adults remains a consequent high risk of HBV transmission and chronic sequelae. Most of the subjects were unknown of their current or former infectious status of HBV because a high proportion of adults seropositive for HBsAg and HBcAb declared that they had not been previously diagnosed with HBV in the interviews. Over 80% of HBV-infected adults do not seek treatment because they are unaware of their illness [20], and the danger of HBV transmission along with the disease burden to the unvaccinated population remains high. Occult HBV infection not only has a great risk of outcomes related to chronic infection, including cirrhosis and hepatocellular carcinogenesis [10], but is also of public health importance as asymptomatic carriers with a high risk of transmitting HBV to others.

It is estimated that 90% of people with HBV infection worldwide have not been diagnosed, which poses a major global health burden that affects 290 million persons globally [21]. Delayed diagnosis resultantly leads to belated identification of cirrhosis, hepatic failure, and HCC, for many patients stay asymptomatic until the onset of end-stage liver disease (ESLD) [22, 23]. One important objective of the survey is to screen out persons with CHB and provide appropriate therapy. The proposed ML algorithms can facilitate “semi-universal” screening for high-risk persons with CHB based on minority demographic characteristics that were easily acquired. Based on the large population and current healthcare infrastructure in China, universal screening would be less cost-effective than risk-based screening in identifying more high-risk people who will benefit from early diagnosis and specific treatment. According to the national progress of the HepB vaccination policy, universal screening is more suitable for adults born before 2002, who missed having the universal birth dose of HepB [24]. Implementation should better consider issues such as who should be screened and where screening should be conducted [20]. Optimal screening could identify most infected individuals with limited resources, and challenges remain in its implementation considering the stigma and discrimination associated with persons with hepatitis B [25].

ML methodology is a core branch of artificial intelligence and is widely applied in the medical field to prioritize population risks of certain diseases [26]. ML-based models have better classification and prediction performance on various medical issues [12, 14, 26, 27] than traditional statistical methods. RF has been found to outperform the logistic regression model and better differentiate higher-risk civilian populations in the United States [14]. Two other machine learning algorithms, SVM and SGB, were creatively applied in the model development process for optimizing HBV screening in field work. We have demonstrated that three variables—age, region, and immunization history—are associated with HBV infection using logistic regression under the circumstance of the present immunization policy. The quantile analysis highlights that the four easily accessed variables (including sex) may be useful in risk stratification in the general population. The three ML models can correctly discover over 70% of individuals with HBV infection in specific demographic groups without requiring persons to disclose illegal or stigmatizing risk behaviors [28].

Limitations of the study include those high-risk behaviors such as blood transfusion, drug injection, tattoos, dental procedures, hemodialysis, … etc., were not investigated, which were shown to be positively associated with HBV infection [15]. Significant features such as birthplace and occupation were not modeled as screening factors due to their extremely unbalanced distribution as a result of the small sample size. Further modification and external validation of models with larger samples are still needed.

## Conclusions

To recapitulate briefly, universal screening is a simple and fair way to identify undiagnosed people with CHB [25]. Integrating targeted screening with other serosurveys or routine physical examinations could help improve cost-effectiveness. Logistic regression combined with ML models could helpfully screen the risk factors for HBV infection and might be utilized to identify high-risk populations with CHB. Regular serological surveys of HBV infection and further screening are instructive and meaningful to understanding the changing context of HepB epidemiology in Chongqing and leading to early diagnosis and treatment of HBV infection. The combined and sustained approach of optimal screening and surveillance strategy, together with earlier initiation of treatment, will be essential to the elimination of HBV transmission in China.

## Data Availability

The datasets used and/or analyzed during the current study are available from the corresponding author on reasonable request.

## Abbreviations

HBV: hepatitis B virus
HBsAg: hepatitis B surface antigen
HBsAb: antibody to HBsAg
HBcAb: antibody to B core antigen
CHB: chronic hepatitis B
HCC: hepatocellular carcinoma
ML: machine learning
RF: random forest
SVM: support vector machine
SGB: stochastic gradient boosting
MTCT: mother-to-child transmission
HepB: hepatitis B vaccine
EPI: expanded program on immunization
WHO: World Health Organization
PPS: probability proportionate to size sampling
CDC: Centers for Disease Control and Prevention
ELISA: enzyme-linked immunosorbent assay
HBIG: hepatitis B immune globulin
ALT: alanine aminotransferase
AST: aspartate aminotransferase
AUC: area under the curve
ROC: receiver operating characteristic curves
ESLD: end-stage liver disease

## References

[1] Organization WH, GLOBAL HEALTH SECTOR STRATEGY ON VIRAL (2016). HEPATITIS 2016–2021.

[2] Meda N, Tuaillon E, Kania D, Tiendrebeogo A, Pisoni A, Zida S (2018). Hepatitis B and C virus seroprevalence, Burkina Faso: a cross-sectional study. Bull World Health Organ.

[3] Liu J, Wang X, Wang Q, Qiao Y, Jin X, Li Z (2021). Hepatitis B virus Infection among 90 million pregnant women in 2853 Chinese counties, 2015–2020: a national observational study. Lancet Reg Health West Pac.

[4] Organization WH, GUIDELINES FOR THE PREVENTION. CARE AND TREATMENT OF PERSONS WITH CHRONIC HEPATITIS B INFECTION. France 2015. 26225396

[5] Muhamad NA, Ab Ghani RM, Abdul Mutalip MH, Muhammad EN, Mohamad Haris H, Mohd Zain R (2020). Seroprevalence of Hepatitis B virus and Hepatitis C virus Infection among Malaysian population. Sci Rep.

[6] Meng J, Xu H, Sui D, Jiang J, Li J, Gao Y (2019). A retrospective serological survey of Hepatitis B virus Infection in Northeast China. BMC Infect Dis.

[7] Liang X, Cui F, Hadler S, Wang X, Luo H, Chen Y (2013). Origins, design and implementation of the China GAVI project. Vaccine.

[8] Wang F, Zheng H, Zhang G, Ding Z, Li F, Zhong G (2015). Effectiveness of prevention of mother-to-child transmission practice in three provinces of Southern China. Hum Vaccin Immunother.

[9] Liang X, Bi S, Yang W, Wang L, Cui G, Cui F (2009). Epidemiological serosurvey of Hepatitis B in China–declining HBV prevalence due to Hepatitis B vaccination. Vaccine.

[10] Cui F, Shen L, Li L, Wang H, Wang F, Bi S (2017). Prevention of Chronic Hepatitis B after 3 decades of escalating Vaccination Policy, China. Emerg Infect Dis.

[11] CM P, DP C (2021). Seroepidemiology of hepatitis A and B in the general population in Hong Kong: protocol of a cross- sectional survey using spatial sampling in a highly urbanised city. BMJ Open.

[12] Zhou J, Song L, Yuan R, Lu X, Wang G (2021). Prediction of hepatic inflammation in chronic Hepatitis B patients with a random forest-backward feature elimination algorithm. World J Gastroenterol.

[13] DeLong ER, DeLong DM, Clarke-Pearson DL (1988). Comparing the areas under two or more correlated receiver operating characteristic curves: a nonparametric approach. Biometrics.

[14] Ramrakhiani NS, Chen VL, Le M, Yeo YH, Barnett SD, Waljee AK (2022). Optimizing Hepatitis B virus screening in the United States using a simple demographics- based model. Hepatology.

[15] Su S, Wong WC, Ong JJ, Seto WK, Zhang L (2022). The optimal screening strategy for chronic Hepatitis B virus Infection in China - authors’ reply. Lancet Glob Health.

[16] Liao XY, Zhou ZZ, Wei FB, Qin HN, Ling Y, Li RC (2014). Seroprevalence of Hepatitis B and immune response to Hepatitis B vaccination in Chinese college students mainly from the rural areas of western China and born before HBV vaccination integrated into expanded program of immunization. Hum Vaccin Immunother.

[17] Xu Y, Liu Y, Wang J, Che X, Zhang X, Jiang W (2021). Hepatitis B virus Infection seromarkers among college freshmen and their immune responses to different vaccination policies of Hepatitis B vaccine. Hum Vaccin Immunother.

[18] Nelson NP, Easterbrook PJ, McMahon BJ (2016). Epidemiology of Hepatitis B Virus Infection and impact of vaccination on Disease. Clin Liver Dis.

[19] Wang AL, Qiao YP, Wang LH, Fang LW, Wang F, Jin X (2015). Integrated prevention of mother-to-child transmission for human immunodeficiency virus, Syphilis and Hepatitis B virus in China. Bull World Health Organ.

[20] Wang C, Cui F (2022). Expanded screening for chronic Hepatitis B virus Infection in China. Lancet Glob Health.

[21] Polaris Observatory C (2018). Global prevalence, treatment, and prevention of Hepatitis B virus Infection in 2016: a modelling study. Lancet Gastroenterol Hepatol.

[22] Abara WE, Qaseem A, Schillie S, McMahon BJ, Harris AM, High Value Care Task Force of the American College of P (2017). Hepatitis B Vaccination, Screening, and linkage to care: best practice advice from the American College of Physicians and the Centers for Disease Control and Prevention. Ann Intern Med.

[23] Nguyen MH, Wong G, Gane E, Kao J-H, Dusheiko G (2020). Hepatitis B Virus: advances in Prevention, diagnosis, and Therapy. Clin Microbiol Rev.

[24] Chang KC, Chang MH, Chen HL, Wu JF, Chang CH, Hsu HY (2022). Universal Infant Hepatitis B Virus (HBV) vaccination for 35 years: moving toward the eradication of HBV. J Infect Dis.

[25] Cohen C, Moraras K, Jackson M, Kamischke M, Gish RG, Brosgart CL (2022). Letter to the editor: importance of universal screening for chronic Hepatitis B Infection in adults in the United States. Hepatology.

[26] Singal AG, Mukherjee A, Elmunzer BJ, Higgins PD, Lok AS, Zhu J (2013). Machine learning algorithms outperform conventional regression models in predicting development of hepatocellular carcinoma. Am J Gastroenterol.

[27] Liu X, Hou Y, Wang X, Yu L, Wang X, Jiang L (2020). Machine learning-based development and validation of a scoring system for progression-free survival in Liver cancer. Hepatol Int.

[28] Conners EE, Panagiotakopoulos L, Hofmeister MG, Spradling PR, Hagan LM, Harris AM (2023). Screening and testing for Hepatitis B Virus Infection: CDC recommendations-United States, 2023. MMWR Recomm Rep.

